# Patient satisfaction for telemedicine health services in the era of COVID-19 pandemic: A systematic review

**DOI:** 10.3389/fpubh.2022.1031867

**Published:** 2022-12-16

**Authors:** Karuna Nidhi Kaur, Farah Niazi, Ruchi Thakur, Shazina Saeed, Shweta Rana, Harpreet Singh

**Affiliations:** ^1^Laboratory of Disease Dynamics and Molecular Epidemiology, Amity Institute of Public Health, Amity University, Noida, India; ^2^Amity Institute of Public Health, Amity University, Noida, India; ^3^Division of Biomedical Informatics (BMI), Indian Council of Medical Research, New Delhi, India

**Keywords:** telehealth, COVID-19, patient satisfaction, health care delivery, telemedicine

## Abstract

**Introduction:**

In the year 2019, the whole world witnessed the COVID-19 pandemic. The pandemic has negatively impacted the health care delivery system. This has risen the necessity among health systems across the world to deliver health care services through telemedicine. This systematic review would assess the level of patient satisfaction with telemedicine health services during the time of the COVID-19 pandemic.

**Methodology:**

The literature search was conducted in June 2022 using “PubMed” “Google Scholar” and “Embase” databases. A total of eight articles were included. ROBVIS Analysis was performed for the assessment of bias. Descriptive statistics were performed using Microsoft Excel.

**Results:**

All included studies were conducted in seven countries/states/cities: India (*n* = 2), Philippines (*n* = 1), Saudi Arabia (*n* = 1), UAE (*n* = 1), Los Angeles (*n* = 1), Iran (*n* = 1), and New York City (*n* = 1). Most used telemedicine tools were voice calls, video calls and messaging/email. Maximum patients used video for consultation (5 out of 9 studies) followed by voice call (4 out of 8 studies), messaging/emails (2 out of 8 studies) and other telemedicine Apps (2 out of 8 studies). Overall, the level of satisfaction was found highest amongst studies conducted in developed countries/states/cities such as New York City (94.9%), Los Angeles (82.7%), UAE (81%) and Saudi Arabia (77.9%) in contrast to studies conducted in developing countries which includes Philippines (82%), India (73.9; 51.3%) and Iran (43.4%).

**Conclusion:**

Most of the participants were found to be satisfied with the quality of telemedicine they were offered. This systematic review will help to improve telemedicine services which will eventually improve the health care delivery system.

**Systematic review registration:**

https://www.crd.york.ac.uk/prospero/#myprospero.

## Introduction

As the COVID-19 pandemic grew internationally, a paradigm shift was seen in the health care delivery system from the traditional way of providing health services which are in-person visits or face-to-face consultations to telemedicine services. According to the WHO, telemedicine is defined as “the delivery of health care services by all health care professionals using technology for the exchange of valid information for the diagnosis, treatment, and prevention of disease and injuries” (1). After the advent of the internet in the 20th Century, many mediums have opened up to provide medical information. However, the use of telemedicine got revolutionized furthermore during the pandemic to reduce the risk of exposure to most people. Many hospitals and clinics across the world are using telemedicine for providing primary care, mental health, obstetrics, gynecology, and many other specialities to reduce exposure to risk during a pandemic (2).

Telemedicine plays an important role in providing continuity of routine care to patients during a pandemic while reducing their exposure risk by minimizing unnecessary visits to hospitals. Patients who were suffering from chronic conditions such as diabetes and hypertension shifted from In-patient to virtual doctors' consultations and follow-up meetings. In this way, digital health services provide compliance with treatment and prevent the severity of the condition. Some of the other advantages of telemedicine are that it is favorable for both providers and patients as it limits non-essential exposure to health care settings, which reduces the spread of infection and delivers clinical information and permits consultation and discussion between health care professionals and patients regardless of where the patient is located, which saves time, reduces travel expenses, and provides easier access without disrupting daily responsibilities. It also helps to generate e-medical records that can be useful later on (3).

In developed countries like the United States (US), most clinical specialities are providing services *via* telemedicine. At present, there are over 200 telemedicine networks, with more than 3,500 services. Data from the Centers for Medicare and Medicaid Services showed an increase in weekly telemedicine visits from 13,000 pre-COVID to 1.7 million visits in the week of April 2020. Compared with data from 2019, telemedicine visits in October 2020 increased by more than 3,000%. This extensive use of telemedicine occurred across nearly all medical specialities. As compared to other countries like the European Union, Japan and Korea, the USA is using telemedicine services at much higher rates. Similarly, in Japan, health care providers and patients are intensively using digital health care facilities. Japan's Health Ministry reported that overall, 10,000 clinics are offering telemedicine services to Japanese people (4, 5).

Increased utilization of telemedicine services has also been seen in developing countries. For instance, in South Africa before the COVID-19 pandemic, telemedicine utilization was limited due to a lack of remuneration. However, since COVID-19, there has been a significant increase in telemedicine utilization. Data showed that in South Africa, tele-triage has reduced the burden on health facilities by saving 95% of face-to-face consultations, and in general practice services, 80% of the problems are resolved by teleconsultation facilities (4). India has also adopted “Telemedicine practice guidelines” approved by the Indian Ministry of Health and Family Welfare on March 25, 2020. It includes the principles and practical framework guidelines for telemedicine in India. After the approval of the guidelines, there has been exorbitant use of telemedicine facilities which can be utilized by various platforms such as telephones, videoconferences, text messaging, emails, and other telemedicine applications (6).

Given the success of telemedicine health services in testing times of the pandemic, there has been an increase in the demand for telemedicine services globally. Hence, it is important to assess patient satisfaction levels for those who have benefitted from it and to identify any lacune that can be dealt with. To summarize all the literature that is available in this aspect of health care delivery, we conducted a systematic review to measure patient satisfaction.

## Methods

### Protocol registration methods

This systematic literature review followed the Preferred Reporting Items for Systematic literature reviews and Meta-Analyses (PRISMA) 2020 guideline has been registered with the Prospective Register of Systematic Reviews (PROSPERO) (Registration ID-343427, https://www.crd.york.ac.uk/prospero/#myprospero).

### Search strategy

The PRISMA guidelines were used to design the methodology for this systematic review (7). The PRISMA statement includes a 27-item checklist which assures transparency, iteration, and complete reporting for systematic reviews. The literature search was conducted in June 2022 using “PUBMED “Google Scholar” and “Embase” databases with the combination of the following term sequences;—Telemedicine, COVID-19, Patient Satisfaction, and Health care delivery Table 1. Manual search in web-based resources was accomplished on Google, journals which published key articles and through searching specific websites like WHO, https://www.who.int, Centers for Disease Control and Prevention (CDC), https://www.cdc.gov, In addition, we reviewed the references of the selected articles in order to identify additional studies or reports not retrieved by the preliminary searches.

**Table 1:** Inclusion and exclusion criteria.

**Inclusion criteria**	**Exclusion criteria**
Original articles assessing the patient satisfaction level with the use of telehealth.	Studies not evaluating the patient's satisfaction with use of telemedicine.
Both cross sectional and cohort study designs were included.	Studies focusing on specific disease like neurological disorder, ophthalmological disorder.
Studies published from January 2020 (advent of COVID-19) to May 2022.	Review articles, case report, case series, commentary, and correspondence were excluded.
Full text articles published in English language.	Studies not published in English language.

### Study selection

**Figure 1:**
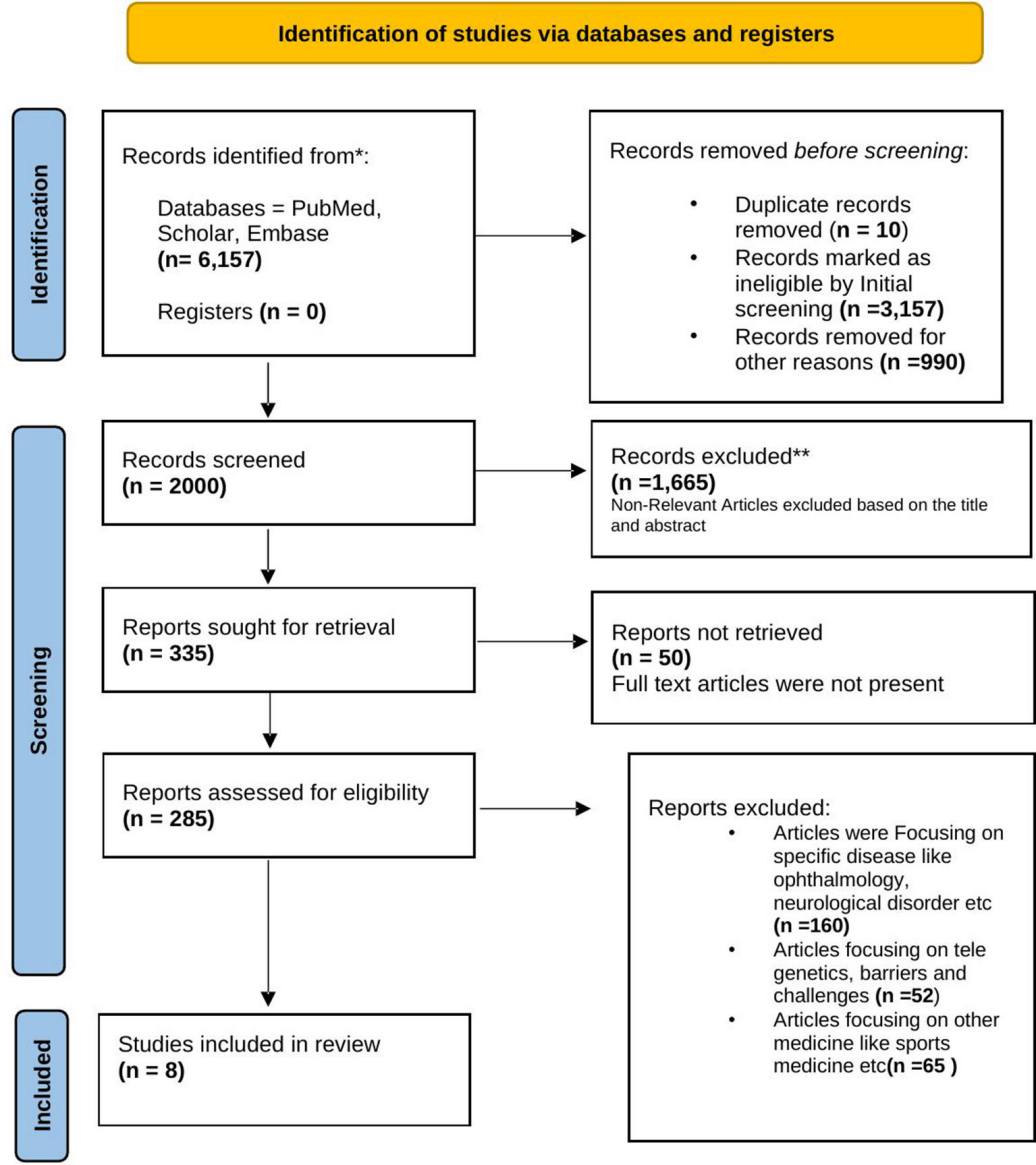
PRISMA diagram.

Two independent researchers selected the relevant studies in a two-step process. In the first step, we reviewed the title and abstracts of the studies for potential eligibility. For the second step, the full texts of these were obtained for in-depth evaluation. Only articles that had reported the level of patient satisfaction and telemedicine during covid19 were included. The initial database search yielded 6,157 published articles matching the keywords over the last 2 years. After eliminating 10 duplicate articles, 3,157 studies were excluded either because of non-English publications or ineligible articles. Furthermore, 990 articles which were not relevant to telemedicine were also removed. After then, 2,000 articles were screened by a researcher who read either the abstracts or full text, which eliminated another 1,665 articles. Articles that were focusing on telemedicine services used for specific diseases like ophthalmology and neurological disorder (*n* = 160) were also excluded, articles focusing on other aspects of telemedicine like tele-genetics (*n* = 52), and articles focusing on other medicine like sports medicine (*n* = 65) were also excluded. After the final screening and evaluation, 8 articles that met the inclusion criteria were finally included as shown in Figure 1.

### Data extraction

The data were extracted and collated into Microsoft Excel spreadsheets and the following domains of data were extracted from each study: study design; participants' information (age, gender), sample size, country/State/City, telemedicine tools, Telemedicine tools, outcome measures and findings. Studies were categorized based on the dimensions of satisfaction results compared. Given the nature of the review question, a meta-analysis was not undertaken and instead a descriptive synthesis and thematic analysis were done.

### Risk of bias assessment

The risk of publication bias was assessed by using the R-based Robvis software package introduced by the National Institute for Health Research (NIHR) (8).

Eight articles were considered eligible for this systematic review. The selection cycle is in accordance with the PRISMA guidelines and is represented as a flow chart in Figure 1.

**Figure 2:**
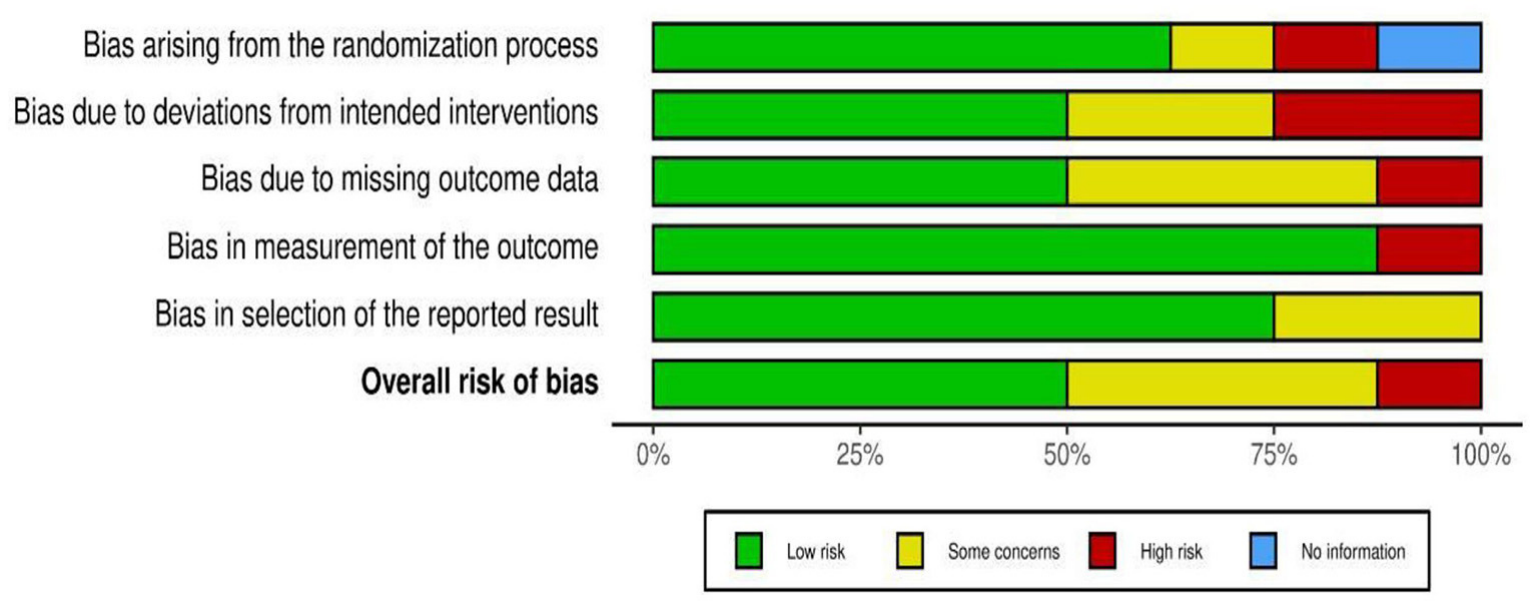
Robvis output for risk bias assessment.

**Figure 3:**
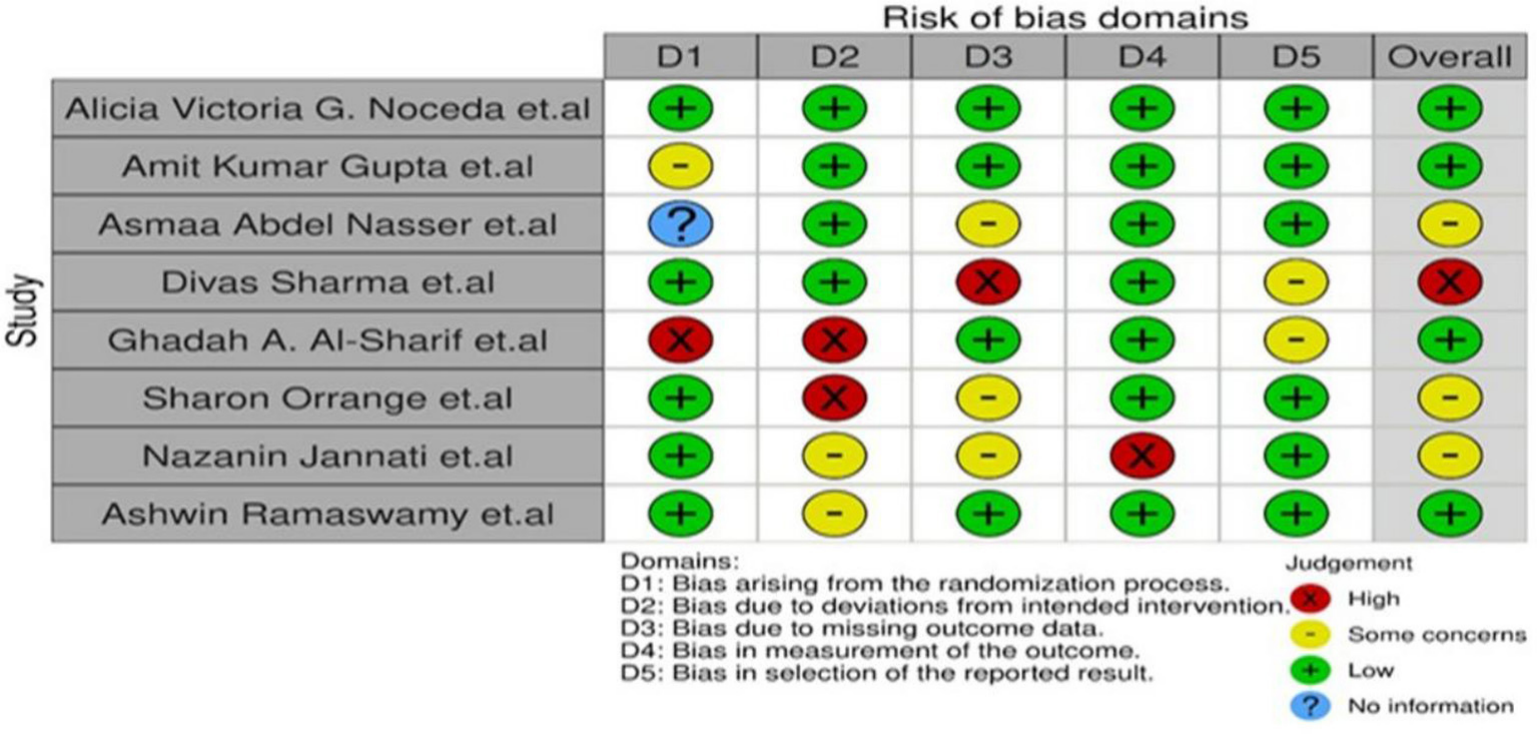
Weighted output for risk bias assessment.

Based on visual inspection of the figure generated by the Robvis software package, there is no potential bias in this study assessing the effectiveness of low-level laser treatment used in various RCT's for TMD patients (Figures 2, 3). Out of 8 studies, 7 (87.5%) are high methodological studies, which have an overall low risk of bias or with some concerns, while only 1 study has a high risk of bias. A detailed description of the eligible studies is given in Table 2.

**Table 2:** Detail characteristics of study included in the review.

**S. no.**	**Name of author**	**Title of study**	**Year**	**Study design**	**Age (years)**	**Sample size**	**Study clinical description detail (%)**	**Country/state**	**Telemedicinetool**				**Outcomemeasures**	**Patient satisfaction (%)**
									**Voice call (%)**	**Video call (%)**	**Messaging/email (%)**	**Telemedicine app (%)**		
1.	Noceda et al. (9)	The patient satisfaction with telemedicine in the Philippines during the covid-19 pandemic	2022	Explanatory mixed method design	18-65	200	Not mentioned	Philippines	31.5	45.5	88.5	23	5-point Likert scale	82
2.	Gupta et al. (10)	Patient's experience of telemedicine during covid-19 pandemic in a tertiary care centre in North India: a telephonic survey	2021	Cross sectional study	≥18	462	Not mentioned	North India (Uttar Pradesh)	100	–	–	–	5-point Likert scale	51.3
3.	Nasser et al. (11)	Measuring the patients' satisfaction about telemedicine used in Saudi Arabia during covid−19Pandemic	2021	Cross sectional study	≥18	425	-Chronic diseases:30.4	Saudi Arabia	–	100	–	–	5-point Likert scale	77.9
							-Cases needing surgical inquires: 5.2							
							-Psychological diseases :4.7							
							-Sexual disease:0.2							
							-Skin diseases :0.7							
							-infectious diseases:2.4							
							-Critical cases needing ICU:12							
							-Dental caries:2.6							
							-Ophthalmology cases: 0.7							
							-Oby/Gyn. Cases:4							
4.	Sharma et al. (12)	Views and Perception on telemedicine by consumers in Delhi	2021	Cross-sectional study	18-39	112	Not mentioned	India (Delhi)	–	–	–	73.19	10-point Likert scale is used to assess satisfaction rate	73.2
5.	Al-Sharif et al. (13)	Telehealth to the rescue during covid-19: A convergent Mixed Method Study Investigating Patients ‘perception	2021	Convergent mixed method study	Not mentioned	100	-Family medicine:22	UAE	22.3	77.7	–	–	Qualitative analysis-thematic analysis	81
							-Internal medicine:16							
							-Neurology:10							
							-Endocrinology:7							
							-Pediatrics:7						Quantitative analysis-SPSS software	
							-Oncology:6							
							-Gastroenterology:5							
							-Rheumatology:5							
							-Obstetrics and Gynecology:4							
							-Orthopedics:3							
							-Cardiology:3							
							-Pulmonology:2							
							-Surgery:2							
							-Ophthalmology:2							
							-Psychology:1							
							-Psychiatrics:1							
							-Nephrology/kidney:1							
6.	Orrange et al. (14)	Patient satisfaction and trust in telemedicine during the covid-19 pandemic: Retrospective Observational study	2021	Retrospective observational study	≥18	368	Not mentioned	Los Angeles	22.6	77.4	–	–	5-point Likert scale	82.7
7.	Jannati et al. (15)	A cross-sectional online survey on patients' satisfaction using store-and-forward voice and text messaging teleconsultation	2021	Cross sectional survey	14-69	396	Not mentioned	Iran	–	–	100	–	5-point Likert scale	43.4
8.	Ramaswamy et al. (16)	Patient satisfaction with telemedicine during the covid-19 pandemic: Retrospective cohort study	2020	Retrospective cohort study	18-80	620	-Internal medicine:47.27	New York	–	100	–	–	5-point likert scale	94.9
							-Obstetrics/ gynecology:17.88							
							-Cardiology:15.31							
							-Ophthalmology :14.05							
							-Otolaryngology :12.89							
							-Hematology/oncology :10.42							
							- Dermatology:8.14							

## Results

Total of 8 studies were included. All included studies were conducted in seven countries/states/city: India (*n* = 2), Philippines (*n* = 1), Saudi Arabia (*n* = 1), UAE (*n* = 1), Los Angeles (*n* = 1), Iran (*n* = 1), and New York City (*n* = 1). Based on the designs of the study, 4 studies were cross-sectional studies, 2 were retrospective studies and 2 were mixed-method study design. Most of the study participants belonged to the age group of ≥18 years. Out of 8 studies, the minimum sample size was 100, considered in mixed method study conducted in UAE and maximum sample size was 620, considered in retrospective cohort study conducted New York City. 3 out of 8 studies reported clinical description details, a study conducted in Saudi Arabia reported 30.4% of the participants believed chronic disease were most suitable diseases that could be managed by telemedicine services. A study conducted in UAE mentioned 22% participants took consultation in family medicine department, 16% in internal medicine department and 10% in neurology department. Another study conducted in New York City reported maximum patients consulted in internal medicine department (47.3%) followed by gynecology department (17.9%) and cardiology department (15.3%).

Most used telemedicine tools were voice call, video call and messaging/Email. Maximum patients used video for consultation (5 out of 9 studies) followed by voice call (4 out of 8 studies), messaging/Emails (2 out of 8 studies) and other telemedicine Apps (2 out of 8 studies). Overall, level of satisfaction was found highest amongst studies conducted in developed countries/states such as New York City (94.9%), Los Angeles (82.7%), UAE (81%), and Saudi Arabia (77.9%) in contrast to the studies conducted in developing countries which includes Philippines (82%), India (73.9%; 51.3%), and Iran (43.4%).

### Tools for telemedicine

**Figure 4:**
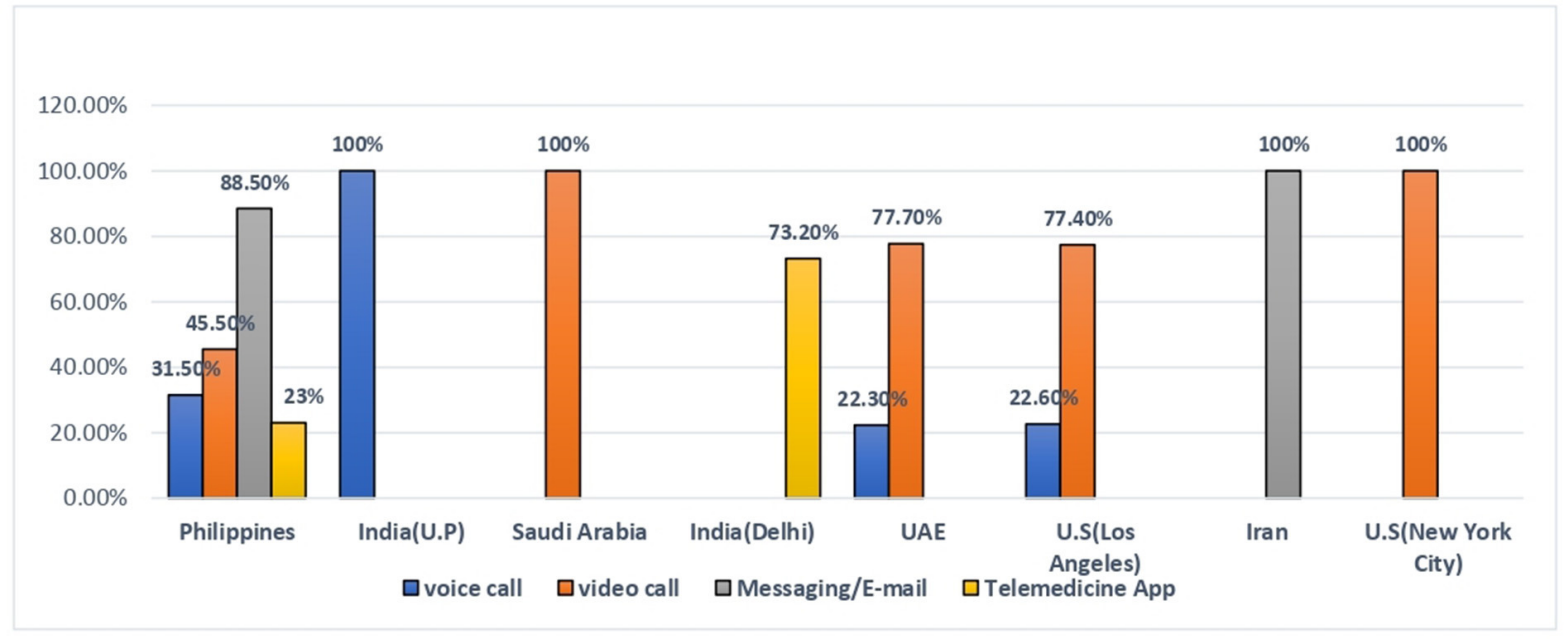
Tools for telemedicine.

Out of 8 studies, the majority of participants used video call, voice call and email/messaging for teleconsultation. Amongst all included studies, study conducted in North India (U.P) revealed maximum participants (100%) used voice call as a telemedicine tool followed by Philippines (31.50%), Los Angeles (22.6%), and UAE (22.3%). Similarly, study conducted in Saudi Arabia and New York City reported highest percentage of participants (100%) took video consultation followed by UAE (77.7%), Los Angeles (77.4%), and Philippines (45.5%). Study findings of Philippines and Iran showed majority (100%) of the participants chose messaging/email for medical advice and study conducted in India (Delhi) reported more than half of the participants (73.2%) used other telemedicine apps (Figure 4).

### Overall patient satisfaction

**Figure 5:**
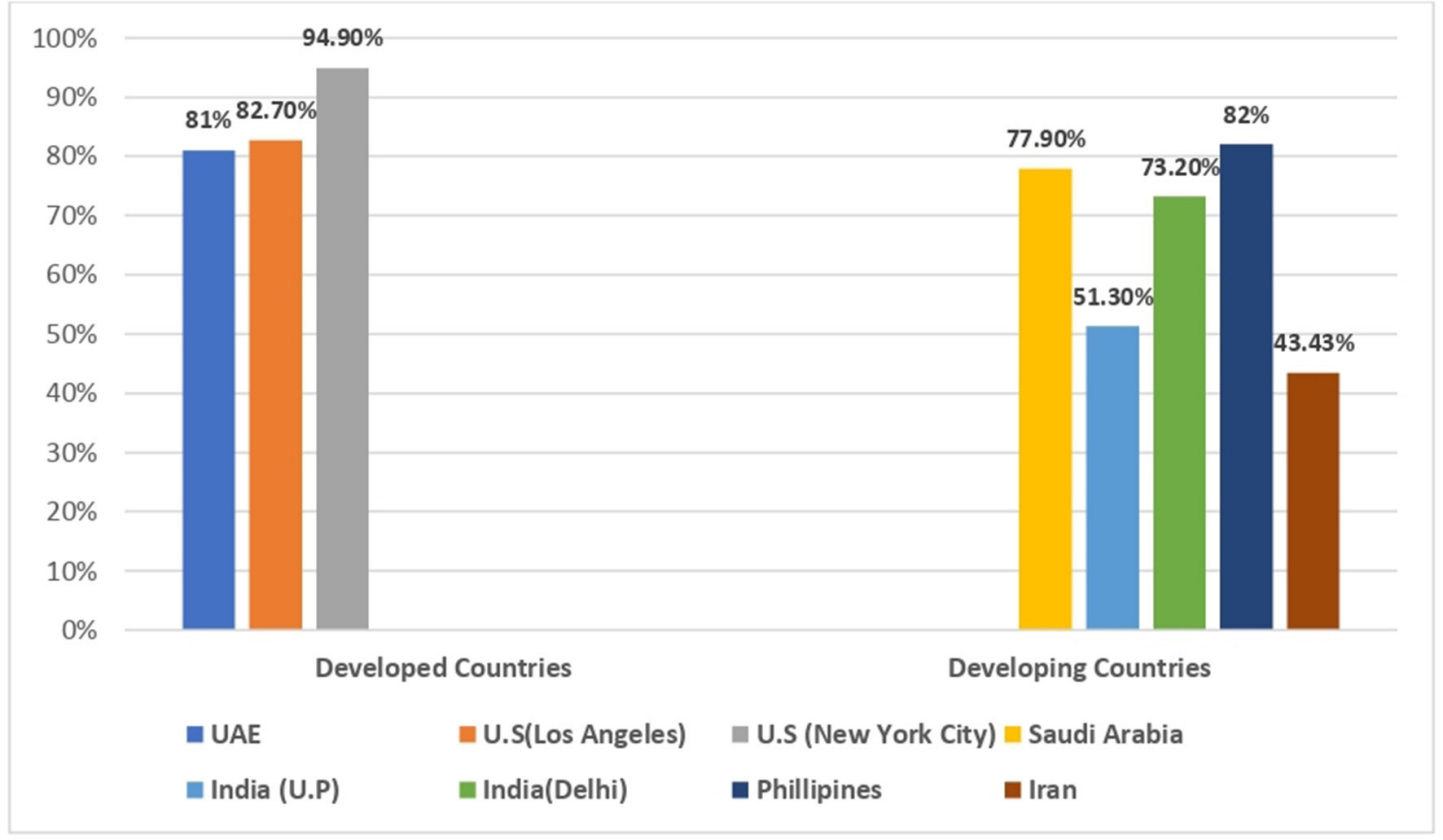
Overall patient satisfaction.

Studies conducted in developed countries/states/City such as New York City (94.9%), Los Angeles (82.7%), UAE (81%), and Iran (43.4%) showed highest satisfaction toward telemedicine services as compared to study conducted in developing countries which includes Philippines (82%), south Africa (77.9%), India; Delhi (73.1%), and North India; U.P (51.3%). Out of Eight studies, Highest participant satisfaction was reported by New York City (94.9%) followed by Los Angeles (82.7%) and Philippines (82%) (Figure 5).

## Discussion

**Figure 6:**
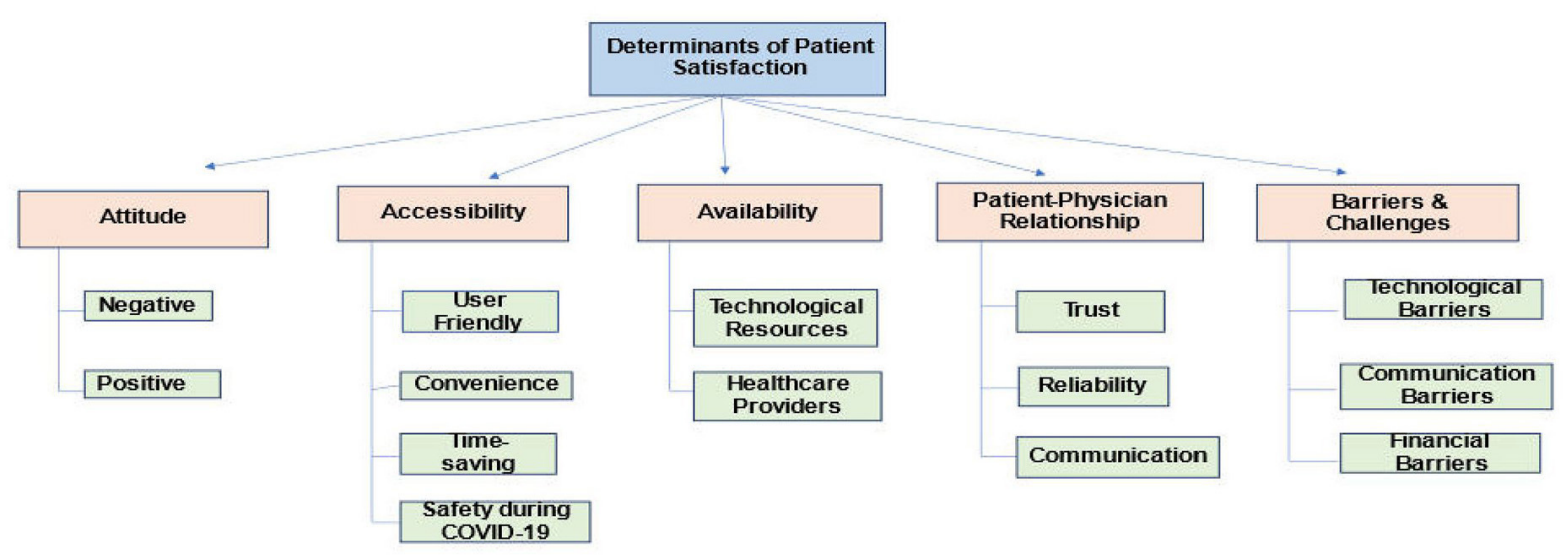
Determinants of patient satisfaction.

Thematic evaluation was conducted for all the 8 studies included in the review. The four themes that were generated are discussed below as depicted in Figure 6.

1. **Evaluation of patient satisfaction rate**.
  Across all the selected studies, most of the patients were generally found to be satisfied with the telemedicine services they received. The satisfaction rate was found higher in studies where video call was used as the medium for teleconsultation (9, 11, 13, 14, 16). In studies where audio call and messaging app were used as the only medium for telemedicine services, the satisfaction rate was found to be relatively lower with a percentage of 51.3 and 43.43 respectively (10, 15). The main reason behind this was the fact that the patients did not find the teleconsultation as similar as a face-to-face consultation (15). Patients belonging to urban areas (54%) and with higher education (53%) were found to be more satisfied than those belonging to rural areas (15). The studies also revealed that the level of patient satisfaction not only depends on the factors that are influencing patient's perception, but also on the qualities exhibited by the physicians or their service providers. This includes trust between patient and physician, consideration exhibited by the health care providers and the expertise of the doctors involved (12, 14).
2. **Accessibility of telemedicine services**.
  Convenience, comfort, ease of access, safety during pandemic and limitation of waiting time, were found to be the major factors influencing the patient satisfaction level toward telemedicine (9–11, 13, 14). Another factor that surfaced was the maintenance of privacy and confidentiality of the patients availing the telemedicine services (13, 15). The patients also admitted that if telemedicine services were not available as an alternative, they might have to miss their work to avail health care services. But with the ease of access to telemedicine, they find it easier to get the required health care without missing out on their work schedule (11).
3. **Barriers and challenges associated with telemedicine services**.
  The main challenges associated with telemedicine services were technological, financial and communication barriers. While majority of the participants were satisfied with the telemedicine services, only 2 in 5 patients found the service to be affordable. According to the patients, they perceived the cost of the telemedicine to be cheaper than in-patient consultation. However, they did not find any difference between the costs of telemedicine and face-to-face consultation and hence were disappointed by this fact (9). The study participants also found the payment methods for telemedicine services to be unnecessarily complicated and confusing. They also faced difficulty with insurance coverage for the telemedicine charges (13). Another major challenge faced by the study participants was the technological issue. Participants found their satisfaction level with the telemedicine services to be dependent on how smoothly a teleconsultation has gone (9). Video and audio quality, stable network connection, and access to technological services were found to be major barriers associated with telemedicine services (9, 11–13). Lack of direct patient-physician interaction was found to be another challenge associated with telemedicine services (10, 12, 13). Lack of trust and confidentiality in health care providers was significantly influenced by the limited direct interaction between users and service providers (9, 10, 13).
4. **Telemedicine implications in a post COVID-19 world**.
  As mentioned previously, one of the major factors for assessment of patient satisfaction level was safety during pandemic. However, most of the participants were found to be satisfied enough to use telemedicine services in the future as well (9, 14). On the other hand, participants recommended further improvement with regards to telemedicine services, such as better network connection, efficient patient-physician communication, ease, and affordability of payment and organized and structured delivery of health care services through telemedicine (12, 13). One study also argued that telemedicine services could only be a supplement to health care services and not a substitute for patient-physician face-to-face consultation. Nevertheless, there is still a need for the wider implementation of the telemedicine services, as it would help in strengthening of health care delivery system for the future pandemics (12).

## Conclusion

This study provided a novel insight for the assessment of the patient satisfaction level with telemedicine. The information extracted from this review will help to delineate the limitations of telemedicine and can be useful in future implementation of telemedicine services in regular practice. Thus, developing a robust and more efficient health care system. The COVID-19 crises posed an important opportunity to strengthen the health care systems, especially for low middle income countries, as telemedicine services are more assessable; affordable and has proven to be a strong public health tool in the future. This systematic review would also be useful for an app developer to design, develop, and implement mobile applications; for health care providers and patients who will use these applications so that points of intervention can be identified and worked upon. This will help to improve telemedicine services which will eventually improve the health care delivery system.

## Data availability statement

The original contributions presented in the study are included in the article/supplementary material, further inquiries can be directed to the corresponding author.

## Author contributions

KK conceptualization and writing. FN methodology design and writing. RT performed the statistical analysis. SS principal investigator, conceptualized, and reviewed the manuscript. SR edited the manuscript. HS supervised and monitored the study implementation. All authors contributed to the article and approved the submitted version.

## References

[1] Polinski JM Barker T Gagliano N Sussman A Brennan TA Shrank WH. Patients' satisfaction with and preference for telemedicine visits. J Gen Internal Med. (2016) 31:269–75. 10.1007/s11606-015-3489-x 26269131 PMC4762824

[2] Perrin PB Pierce BS Elliott TR. COVID-19 and telemedicine: a revolution in health care delivery is at hand. Health Sci Rep. (2020) 3:14. 10.1002/hsr2.166 32500101 PMC7261969

[3] Hebbar PB Sudha A Dsouza V Chilgod L Amin A. Health care delivery in India amid the Covid-19 pandemic: challenges and opportunities. Indian J Med Ethics. (2020) 5:1. 10.20529/IJME.2020.064 32546453 PMC7611350

[4] Omboni S Padwal RS Alessa T Benczúr B Green BB Hubbard I . The worldwide impact of telemedicine during COVID-19: current evidence and recommendations for the future. Connect Health. (2022) 1:7. 10.20517/ch.2021.03 35233563 PMC7612439

[5] Kichloo A Albosta M Dettloff K Wani F El-Amir Z Singh J . Telemedicine, the current COVID-19 pandemic and the future: a narrative review and perspectives moving forward in the USA. Family Med Commun Health. (2020) 8:610. 10.1136/fmch-2020-000530 32816942 PMC7437610

[6] Telemedicine Practice Guidelines. Ministry of Health and Family Welfare. Philippines: Telemedicine Practice Guidelines (2020). Available online at: https://www.mohfw.gov.in/pdf/Telemedicine.pdf (accessed July 10, 2022).

[7] PRISMA. Transparent Reporting of Systematic Review and Meta-analysis. Easley: PRISMA (2021). Available online at: https://prisma-statement.org/ (accessed July 10, 2022).

[8] McGuinness LA Higgins JPT. Risk-of-bias VISualization (robvis): an R package and Shiny web app for visualizing risk-of-bias assessments. Res Syn Meth. (2020) 12:1–7. 10.1002/jrsm.1411 32336025

[9] Noceda A Acierto LM Bertiz MC Dionisio DE Laurito CB Sanchez GA . Patient satisfaction with telemedicine in the Philippines during the COVID-19 pandemic: a mixed methods study. Res Square. 10.1101/2022.05.21.22274939 PMC10032251 36949479

[10] Gupta AK Paul S Soni A Kumar P Nath B Jotdar A. Patient's experience of telemedicine during COVID-19 pandemic in a tertiary care centre in North India: a telephonic survey. Int J Commun Med Public Health. (2021) 8:2517–22. 10.18203/2394-6040.ijcmph20211785

[11] Nasser AA Alzahrani RM Fellah CA Jreash DM Almuwallad NT Bakulka DS . Measuring the patients' satisfaction about telemedicine used in Saudi Arabia during COVID-19 pandemic. Cureus. (2021) 13:e13382. 10.7759/cureus.13382 33754105 PMC7972323

[12] Sharma D Mittal M Pareek M. Views and perceptions on telemedicine by consumers in Delhi. Med Sci. 33. 10.9734/JPRI/2021/v33i57B3403135024509

[13] Al-Sharif GA Almulla AA AlMerashi E Alqutami R Almoosa M Hegazi MZ . Telemedicine to the rescue during COVID-19: a convergent mixed methods study investigating patients' perception. Front Public Health. (2021) 9:9510. 10.3389/fpubh.2021.730647 34917570 PMC8669510

[14] Orrange S Patel A Mack WJ Cassetta J. Patient satisfaction and trust in telemedicine during the COVID-19 pandemic: retrospective observational study. JMIR Hum Fact. (2021) 8:e28589. 10.2196/28589 33822736 PMC8103305

[15] Jannati N Nakhaee N Yazdi-Feyzabadi V Tjondronegoro D. A cross-sectional online survey on patients' satisfaction using store-and-forward voice and text messaging teleconsultation service during the covid-19 pandemic. Int J Med Inform. (2021) 151:104474. 10.1016/j.ijmedinf.2021.104474 33965682 PMC8095037

[16] Ramaswamy A Yu M Drangsholt S Ng E Culligan PJ Schlegel PN . Patient satisfaction with telemedicine during the COVID-19 pandemic: retrospective cohort study. J Med Internet Res. (2020) 22:e20786. 10.2196/20786 32810841 PMC7511224

